# Mapping Complex Interventions in Portuguese Healthcare Research: A Scoping Review Protocol

**DOI:** 10.3390/nursrep12010015

**Published:** 2022-03-01

**Authors:** Filipa Ventura, Odete Araújo, Tiago Casaleiro, Tânia Morgado, Cláudia Oliveira

**Affiliations:** 1 Health Sciences Research Unit: Nursing, Nursing School of Coimbra, 3046-851 Coimbra, Portugal; odete.araujo@ese.uminho.pt (O.A.); cjoliveira@ualg.pt (C.O.); 2 School of Nursing, University of Minho, 4710-057 Braga, Portugal; 3 Centre for Interdisciplinary Research in Health, Institute of Health Sciences, Universidade Católica Portuguesa, 1649-023 Lisbon, Portugal; s-tcasaleiro@ucp.pt; 4 Centro Hospitalar e Universitário de Coimbra—Hospital Pediátrico, Av. Afonso Romão, 3000-062 Coimbra, Portugal; tmorgado@esenfc.pt; 5 School of Health Sciences, Polytechnic of Leiria Portugal, 2411-901 Leiria, Portugal; 6 Universidade do Algarve, 8005-139 Faro, Portugal

**Keywords:** complex interventions, Health Services Research, Portugal, review

## Abstract

Along with the worldwide recognition of the importance of the methodological guidance to the validity and rigour of complex health interventions, this scoping review aims to identify and characterise the scientific evidence on complex health interventions in Portuguese healthcare research. The Joanna Briggs Institute (JBI) guidance for scoping reviews will be followed. The population (P) concept (C) context (C) mnemonic will scaffold the research questions, inclusion and exclusion criteria, and searching strategy. MEDLINE, CINAHL, LILACS, and Open Access Scientific Repository of Portugal (RCAAP) will be searched. Scientific evidence reporting complex health interventions in the Portuguese healthcare context, in Portuguese and English and published from 2008 and onwards will be considered for inclusion. Literature pertaining to complex health interventions outside the Portuguese healthcare context will be excluded. The literature will be screened for eligibility by two independent reviewers first by title and abstract and subsequently by full-text. A data matrix will be used for data-extraction of the included literature. The charted data will be thematically analysed and presented graphically with a narrative description of the literature characteristics. The results from this literature review are expected to provide an overview of the knowledge concerning the characteristics and methodological guidance of Portuguese complex health interventions.

## 1. Introduction

Complex interventions in health are commonly used in the healthcare services and they are provided and evaluated at different levels (e.g., from an individual to social level). The evidence demonstrates that the research of complex interventions in health is essential to promote efficient clinical practice and promote positive impact on healthcare or population health outcomes [1]. From the analysis of clinical practice in different fields, health interventions are consensually considered to be complex, as they entail many components and inter-relational elements that might hamper achieving the envisioned outcomes. Therefore, there are many definitions of complex interventions that are pivotal in the clarification of this concept and in the subsequent research designs used to investigate them.

Over the years, three definitions of complexity emerged. The first definition was focused on characteristics of the interventions delivered [2,3]. Lately, some authors presented a different definition, with the categorization of complexity beyond the components of the intervention [4] and have also considered complexity concerning the implementation (e.g., how an intervention can differ as it is implemented), the context (e.g., the different situations in which an intervention is implemented) and the participants (e.g., the variation in participants getting the intervention). The last definition that appeared comes after the content analyses of the definition of complex interventions in 207 journal articles, and suggests that a complex intervention can be described in terms of interventions, designs, implementation, context, outcomes, and evaluation challenges [5,6]. Regardless of the definition adopted, it is consensual that a complex intervention is much more than the sum of its component parts [5].

Pursuing transparency, the proposed scoping review is underpinned on the definition of complex interventions as activities that contain a number of component parts with the potential for interactions between them which, when applied to the intended target population, produce a range of possible and variable outcomes [5]. Furthermore, scholars have established that all complex interventions have two common characteristics: (i) multiple complements, and (ii) multiple causal pathways. These might be furthered complicated by (i) multiple target groups, (ii) multifaceted uptake strategies, and (iii) dynamic multidimensional context [7]

Given the complexity of the concept itself and the number of components to be considered in this type of research, methods to conduct research on complex health interventions have been established to increase the possibility of the generated knowledge to reach and benefit humanity [5]. In 2000, the United Kingdom Medical Research Council (MRC) published a framework (f) for researchers about how to develop, test, evaluate, and implement complex interventions in health as separate, although non-linear, steps with important feedback loops to previous and succeeding stages [2]. The MRC-f was revised in 2008, where several important conceptual, methodological and theoretical developments have taken place [2,5]. More recently in 2021, the National Institute of Health Research (NIHR) and the MRC updated the framework, according to the most recent developments in systems theory and methods to maximise the efficiency, use, and impact of intervention research [1]. Nowadays, a range of more detailed guidance on specific aspects of the research process accompanies the MRC-f [1].

The goal of the MRC-f is to support researchers to develop an intervention that is acceptable, useful and used in clinical practice, to demonstrate its effect beyond the vagaries of chances, and embed it in clinical situations where it will bring the most benefit to patients, healthcare professionals, and the wider healthcare organisation [5]. This methodological guidance (MRC-f) allows the development of knowledge to improve practice and the quality of the care provided. In this sense, the framework endorses researchers’ work in iterative and cyclic collaboration with stakeholders (i.e., patient and public involvement and engagement), to identify the key complexity elements in health interventions. Generally knowledge is thought of as a collection of facts, information, or description of a phenomenon or a thing. From a health care perspective, knowledge to be applied in practice is expected to be scientifically analysed so as to prevent harm being done to the patient, maximise the outcome of the care delivered and treatment, and guarantee the best use of the organisation’s resources [8].

Furthermore, the MRC-f advocates that the research design and conduct should entail a diversity of methodological perspectives and appropriate choice of methods (i.e., multiple methods approach), while attending to the context where the intervention will unfold from the early-stages of intervention development (i.e., systems’ perspective) [1].

The use of complex interventions in clinical practice, as well as research in this area, has increased in the last years, due to the development, refinement, and dissemination of the framework that supports this methodology. It is important to mention that although the MRC-f is the gold standard for research into complex health interventions, many researchers develop, test, and evaluate complex interventions without the use of this particular guidance, albeit with converging points. The MRC-f is usefully summarised as a four-stage process of “develop-test-evaluate-implement”. At all stages in this process, researchers aim to address the key uncertainties in their intervention, their research design, and their procedural strategies before moving on to reporting the effects of their interventions and working to embed it into routine health care [5]. In brief, methodological guidance has brought consistency to the development, feasibility/piloting, evaluation, and implementation of complex health interventions, particularly by allowing the identification and management of complexity in a stepwise manner across the intervention lifecycle.

A recent literature review, although carried out by Portuguese authors, focused on the international use of MRC-f specifically in the field of nursing research [9]. The review acknowledges the importance of using the specific guidance depicted by the MRC-f in order to develop more feasible and effective nursing interventions. It further identifies lack of time and of financial support, subjectivity of interventions, and sample size constraints, as barriers to the development of such interventions. The significance of having adequate skills to plan and conduct complex interventions research is highlighted. Alongside the update to the MRC-f published in 2021 [1], the proposed literature review will further complement the previous review by studying the reality of Portuguese healthcare research, including unpublished work, thereby providing an overview of research into complex interventions beyond the nursing domain with the potential to enhance the understanding of barriers and facilitators specific to Portuguese healthcare research.

The worldwide recognition of the importance of the methodological guidance to the validity and research rigour of complex interventions reinforces the need to understand its spread and adoption in Portuguese healthcare research. Additionally, an overview of the research may identify gaps in research or overlooked themes.

A preliminary search of PROSPERO, MEDLINE, and the Cochrane Database of Systematic Reviews, was conducted, and no on-going scoping reviews or systematic reviews on complex health interventions in the Portuguese context were identified. This step must be conducted according to the JBI methodology and aims at avoiding the duplication of research products. Given the encouragement to publish and register review protocols of literature reviews, authors should investigate whether there is a review on the pursued topic that is already being conducted prior to initiating their own. Overall the literature review will seek to answer: how are complex interventions being conducted in Portuguese healthcare research? The identified evidence will be specifically characterised by addressing the following specific questions:

- In which health care domains is complex health intervention research being conducted?
- What is the methodological guidance that is followed throughout the intervention research lifecycle?
- How are the complex health interventions designated?
- What are the complexity elements identified?
- What is the theory underpinning the complex health intervention?
- Who are the persons delivering the complex health intervention?
- Who are the persons receiving the complex health intervention?
- What are the contexts of intervention delivery?
- What is the format of intervention delivery?
- What outcomes (process, effectiveness, and implementation) are being assessed?
- What is/are the completed phase(s) of the study in relation to the intervention research lifecycle?

Accordingly, the purpose of this paper is to present a protocol to identify and characterise the scientific evidence on complex health interventions in Portuguese healthcare research.

## 2. Methods

The proposed scoping review will be conducted in accordance with the Joanna Briggs Institute (JBI) methodology for scoping reviews [10] and will adhere to the Preferred Reporting Items for Systematic Reviews and Meta-analyses for Scoping Reviews (PRISMA-ScR) guidelines [11]. Accordingly, the population (P) concept (C) context (C) mnemonic will scaffold the inclusion and exclusion criteria and the design of the searching strategy. The proposed review will consider literature that reports on complex interventions (i.e., concept) carried out by healthcare professionals to healthcare service users (i.e., population) in Portuguese healthcare care settings (i.e., context).

### 2.1. Inclusion Criteria

#### 2.1.1. Population

Healthcare professionals include, but are not limited to, nurses, physicians, psychologists, physiotherapists, nutritionists or midwives. Literature involving non-healthcare professionals will be excluded. Healthcare service users include all persons participating in healthcare processes with healthcare professionals (i.e., seeking or receiving healthcare), which might include, but are not limited to, persons of all ages, patients of all ages, relatives, family, community groups, and older adults residents, irrespective of a disease/illness domain.

#### 2.1.2. Concept

This review will consider studies that explore complex interventions, irrespective of their methodological guidance, as long as they comply with at least two complexity domains of the following [7]:

- Intervention complexity—they have multiple components;
- Pathway complexity—complicated or multiple causal pathways, feedback loops, synergies, and/or mediators and moderators of effect;
- Population complexity—target multiple participants, groups, or organisational levels;
- Implementation complexity—require multifaceted adoption, uptake, or integration strategies;
- Contextual complexity or work in a dynamic multi-dimensional environment.

Additionally, studies reporting on interventions that target a range of behaviours and demand expertise and skills from interventionists and participants will also be considered for inclusion, as these elements have been also identified as complexity elements [1].

Studies will be considered for inclusion irrespectively of their phase in relation to the intervention research cycle, i.e., development, feasibility, evaluation, or implementation. Interventions will be considered for inclusion if identified as, but not limited to, psychosocial, behavioural, cognitive, supportive or educational, and irrespective of the underpinning theory.

Interventions will be considered for inclusion, but are not limited to, if targeting the following outcomes, clinical/health-related, patient-reported (e.g. fatigue, pain, quality of life, self-efficacy, distress) or processual (e.g. adherence to treatment, costs, care continuity, care accessibility). Interventions targeting outcomes not related to the health care sciences domain will be excluded.

#### 2.1.3. Context

The proposed review will consider literature reporting on complex health interventions at all Portuguese healthcare settings irrespective of their healthcare level (i.e., primary, secondary, or tertiary), including community healthcare, or geographical location (i.e., urban, rural). Studies pertaining to non-Portuguese healthcare settings will be excluded.

#### 2.1.4. Types of Sources

This scoping review will consider quantitative, qualitative, and mixed methods study designs for inclusion. Systematic reviews will also be considered for inclusion. Text and opinion papers will not be considered for inclusion in the proposed scoping review.

### 2.2. Search Strategy

The search strategy will aim to locate both published and unpublished primary studies. An initial limited search of MEDLINE (PubMed) was undertaken to identify articles on the topic. The text words contained in the titles and abstracts of relevant articles, and the index terms used to describe the articles were used to develop a full search strategy for MEDLINE (PubMed), CINAHL (EBSCO), Scielo, and LILACS. Sources of unpublished studies to be searched include the Open Access Scientific Repository of Portugal (RCAAP). The search strategy, including all identified keywords and index terms, will be adapted for each included information source. The reference lists of articles included in the review will be screened for additional papers. Articles published in Portuguese or English will be included. Articles published from 2008 to the present will be included, as this represents the publishing year of the MRC-f as the most disseminated methodological guidance for complex health interventions [2].

As recommended by the JBI methodology, we built and tested our search strategy (c.f. Table A1) in Medline (PubMed) and a total of 371 references were retrieved matching complex interventions carried out by healthcare professionals to healthcare service users in Portuguese healthcare care settings. As Scielo and LILACS will also be searched for being more oriented ibero-american studies, we expect the preliminary reference count to be even greater than the presented.

For the current review, searching the main literature reviews registries (i.e., Cochrane and Prospero) and Medline for published scoping review protocols in the topic, no result could be found. This fact endorses the pursuance of the scoping review.

### 2.3. Study/Source of Evidence Selection

Following the search, all identified records will be collated and uploaded into EndNote 20 (Clarivate Analytics, Philadelphia,, PA, USA) and duplicates removed. Following a pilot test, titles and abstracts will then be screened by two independent reviewers for assessment against the inclusion criteria for the review. Potentially relevant papers will be retrieved in full and their citation details imported into the Rayyan. The full text of the selected citations will be assessed in detail, against the inclusion criteria by two independent reviewers. Reasons for exclusion of full-text papers that do not meet the inclusion criteria will be recorded and reported in the scoping review. Any disagreements that arise between the reviewers at each stage of the selection process will be resolved through discussion or with a third reviewer. The results of the search will be reported in full in the final scoping review and presented in a PRISMA-ScR flow diagram [11].

### 2.4. Data Extraction

Data will be extracted from papers included in the scoping review by two independent reviewers using a data extraction tool developed by the reviewers. The data extracted will include specific details about the healthcare professionals delivering the complex health intervention, as well as the individuals receiving it, the characteristics of the complex interventions, the specificities of the Portuguese healthcare settings, and other key findings relevant to the review question. A draft extraction tool is provided (see Table A2). The draft data extraction tool will be modified and revised as necessary during the process of extracting data from each included paper. Modifications will be detailed in the full scoping review. Any disagreements that arise between the reviewers will be resolved through discussion or with a third reviewer. Authors of papers will be contacted to request missing or additional data, where required.

### 2.5. Data Analysis and Presentation

The charted data will be synthesised and thematically analysed [10] following the data extraction domains relating to the characteristics of healthcare professionals, individuals, and contexts of the intervention research developed in Portuguese healthcare contexts, as well as the elements of the included complex health interventions, with a narrative description of the literature characteristics.

The results will be presented in a tabular and graphical form supplemented with a narrative summary that relates them to the review’s objective and specific research questions.

## 3. Discussion

The proposed scoping review will aim to identify and characterise the scientific evidence on complex health interventions in Portuguese healthcare research, following the JBI methodology for scoping reviews.

Given the many elements of complexity behind health interventions, methodological consensus might be difficult to reach. Although intervention research is increasingly advancing methods to manage uncertainties throughout the different stages of development, feasibility, evaluation, and implementation, tackling these uncertainties is no one-researcher endeavour. The methodological heterogeneity demands even greater awareness in relation to the research problem, the context, and the priorities and expectations of the persons involved in the intervention, that is to say, patients and healthcare providers. The challenges are manifold and only collaborative, multidisciplinary work can successfully allow us to develop, test, evaluate, and implement a health intervention that is usable, useful, and used in clinical daily practice.

The results from this literature review are expected to provide an overview of the knowledge concerning the characteristics and methodological guidance of Portuguese complex health interventions. Understanding how the challenges portrayed in the international literature are emerging and being managed in the Portuguese healthcare context is essential to identify strengths and weaknesses, which might be adequately addressed thereafter. Focusing on the whole process of Portuguese complex health interventions, i.e., from design to implementation, and the challenges that might rise in that process, might allow one to identify areas where specific training is required and highlight pros and cons of following a systematic and rigorous approach to intervention development, testing, evaluation, and implementation. Local training, nation-wide supportive peer-researcher networks, and articulation with European communities of practice (e.g., European Academy of Nursing Science) might be important towards enhancing collaborative work in complex health interventions, and thereby the research rigour and the validity of the intervention.

In terms of context-level, the proposed scoping review will mostly likely retrieve evidence from the micro-level (i.e., clinical contexts, nearest to the patient-healthcare team encounter). Even though the updated methodological guidance is approaching twelve years since its first European release, studies involving the meso-level (i.e., healthcare organisation) and macro-level (i.e., Portuguese healthcare system) that follow the MRC-f guidance are likely to be found towards the end of the intervention research cycle, which might take several years to reach.

Departing from the searching strategy built upon the inclusion/exclusion criteria, we expect that the current scoping review will retrieve evidence of which outcomes are evaluated, which evaluation methods (e.g., realistic evaluation), and which strengths and challenges researchers are facing in that evaluation process. It is expected that the scoping review may be a precursor of systematics reviews, such as a review of the effectiveness of the interventions.

Beyond the evaluation phase, we expect similar evidence concerning the design/development, feasibility, and implementation phases. Additionally, methodological aspects will be analysed and difficulties to adhere to the framework may arise. The characterization of the identified studies will reveal which phases of the framework were performed, allowing the identification of gaps in the use of the methodology.

## 4. Conclusions

The proposed scoping review will aim to identify and characterise the scientific evidence on complex health interventions in Portuguese healthcare research, following the JBI methodology for scoping reviews.

Overall, the scoping review will provide a comprehensive overview of the state-of-the-art of complex health interventions in Portuguese healthcare contexts. Upon the comprehensive review, a discussion of the findings to support Portuguese complex health interventions with researchers will be conducted towards enhancing the rigour of intervention research processes aiming at acceptable, feasible, and effective interventions that are successfully adopted in clinical practice.

## Appendix A

**Table A1:** Draft of search strategy to Medline (PubMed) ^1^.

Search No.	Query	Records Retrieved
#1	((((((((health personnel[MeSH Terms]) OR (“health professional”[Title/Abstract])) OR (“healthcare professional”[Title/Abstract])) OR (physician[Title/Abstract])) OR (nurse[Title/Abstract])) OR (physiotherapist[Title/Abstract])) OR (nutritionist[Title/Abstract])) OR (psychologist[Title/Abstract])) OR (midwife[Title/Abstract])	827,852
#2	(((((((Humans[MeSH Terms]) OR (Patients[MeSH Terms])) OR (Persons[MeSH Terms])) OR (relative[Title/Abstract])) OR (family[Title/Abstract])) OR (individuals[Title/Abstract])) OR (client[Title/Abstract])) OR (subject[Title/Abstract])	21,125,847
#3	((((((Research Design*[MeSH Terms]) OR (“complex intervention”[Title/Abstract])) OR (“intervention program*”[Title/Abstract])) OR (intervention[Title/Abstract])) OR (“intervention approach”[Title/Abstract])) OR (“health intervention”[Title/Abstract])) OR (intervention[Title/Abstract])	1,136,000
#4	((((Health services*[MeSH Terms]) OR (“health institution”[Title/Abstract]) OR (“healthcare institution”[Title/Abstract]))) OR ((“Primary Health Care”[Mesh]) OR (“Ambulatory Care Facilities”[Mesh])) OR ((“Assisted Living Facilities”[Mesh]) OR (nursing homes[Title/Abstract])) OR (hospital*[Title/Abstract]))	5,686,278
#5	((“Portugal”[MeSH Terms]) OR (“Portugal”[All Fields])) OR (“Portuguese” [All Fields]) OR (Portug*[All Fields])	239,152
#6	#1 AND #2 AND #3 AND #4 AND #5	371

^1^ Search date: 12 December 2021.

## Appendix B

**Table A2:** Draft of data extraction matrix.

Main Field	Extraction Categories	Category Description
Study ID	1. Reference number	
	2. Authors	
	3. Year	
	4. Title	
	5. Journal	
	6. Issue no.	
	7. Vol no.	
	8. Type of reference	1. Primary research
		2. Systematic review
		3. Unpublished research
Inclusion/Exclusion criteria	9. Does the literature involve healthcare professionals?	1: Yes
		2: No
	10. Does the study involve research about a complex health intervention?	1: Yes
		2: No
	11. Does the intervention target outcomes related to the healthcare sciences domain?	1: Yes
		2: No
	12. Does the study occur in a Portuguese healthcare context?	1: Yes
		2: No
	13. Does the study pertain to primary research, systematic review or unpublished research?	1: Yes
		2: No
	14. Are there any other reasons for exclusion?	1: Yes
		2: No
	15. Inclusion of paper	1: Yes
		2: No
Characteristics of population	16. Who are the healthcare professionals in the healthcare practice/intervention?	1. Physicians
		2. Nurses
		3. Psychologists
		4. Physiotherapists
		5. Psychologists
		6. Nutritionists
		7. Midwifes
		8. Multidisciplinary
		9. Other, please specify
	17. Who are the healthcare service users involved in the healthcare practice/intervention?	1. Persons of all ages
		2. Patients of all ages
		3. Relatives
		4. Family
		5. Community groups
		6. Residents
		7. Other, please specify
Characteristics of Complex health interventions	18. In which health care domains is complex health intervention research being conducted?	1. Medicine
		2. Nursing
		3. Psychology
		4. Multidisciplinary, please specify
		5. Other, please specify
	19. What is the methodological guidance applied?	1. Intervention research framework
		2. Not explicitly stated
	20. What is the theory, model, and framework guiding the complex health intervention?	1. Please describe
		2. Not explicitly stated
	21. How is the complex health intervention designated?	1. Educational
		2. Psychosocial
		3. Supportive
		4. Communicational
		5. Other, please specify
	22. What is the type of complexity identified?	1. Intervention complexity
		2. Pathway complexity
		3. Population complexity
		4. Implementation complexity
		5. Contextual complexity
		6. Interventionist complexity
		7. Multiple behaviours
		8. Other, please specify
	23. What is the format of intervention delivery?	Please describe
	24. What are the outcomes being assessed?	1. Effectiveness, please specify
		3. Process, please specify
		2. No outcomes are assessed
	25. What is/are the completed phase(s) of the study in relation to the intervention research lifecycle?	1. Development
		2. Feasibility
		3. Piloting
		4. Evaluation
		5. Implementation
		6. Not explicitly stated
		7. Other relevant information
Characteristics of context	26. What is the setting of intervention delivery?	1. Healthcare setting
		2. Community setting
		2. Person’s home
		3. Virtual
		4. Other, Please specify
Literature summary		
Reviewers commentaries		
